# Acute Esophageal Necrosis: A Case Series

**DOI:** 10.7759/cureus.2391

**Published:** 2018-03-29

**Authors:** Leon D Averbukh, Marianna G Mavilia, Grigoriy E Gurvits

**Affiliations:** 1 Internal Medicine, University of Connecticut Health Center; 2 Gastroenterology, Nyu-Langone

**Keywords:** acute esophageal necrosis, gurvits syndrome, black esophagus, esophagus, gastroesophageal junction

## Abstract

Acute esophageal necrosis (AEN) is a particularly rare syndrome with an incidence of only 0.1-0.28%, whose appearance is notable for proximal extensions of black, necrotic appearing mucosa extending proximally in the esophagus and abruptly interrupted at the gastroesophageal junction. In this case series, we explore the cases of two males: one middle-aged and one elderly, who after presenting with emesis, were found to have acute esophageal necrosis on esophagogastroduodenoscopy.

## Introduction

Acute esophageal necrosis (AEN), also known as Gurvits syndrome or black esophagus, is a particularly rare syndrome with an incidence of only 0.1-0.28% and a 4:1 predilection to males [1]. It has a striking endoscopic appearance and is notable for nearly universal involvement of the distal esophagus with various proximal extensions of black necrotic appearing mucosa and abrupt interruption at the gastroesophageal junctions (GEJ) [2]. The degree of tissue penetration is variable and likely related to the severity of the insult [3-4]. Over 90% of patients with AEN present with signs of upper gastrointestinal (GI) hemorrhage, including hematemesis, coffee-grounds emesis, melena, and blood loss anemia [1,5]. Associated conditions may include cardiovascular compromise, shock, diabetic ketoacidosis, vasculopathy, aortic dissection, alcohol intoxication, thromboembolic phenomena, malignancy, duodenal ulcer disease, gastric outlet obstruction, hiatal hernia, and malnutrition [1,6-7]. In this case series, we explore two cases of critically ill patients in whom AEN was documented on esophagogastroduodenoscopy (EGD) and discuss the likely pathophysiology of the condition.

## Case presentation

Case 1

A 79-year-old male presented to our institution with repeated episodes of coffee-grounds emesis. He denied non-steroidal anti-inflammatory drug (NSAID) use and had no history of prior GI bleeding. His past medical history was notable for chronic obstructive pulmonary disease, hypertension, congestive heart failure, bladder cancer, gastroesophageal reflux disorder, and end-stage renal disease on peritoneal dialysis. His regular medications included low-dose aspirin, atorvastatin, budesonide nebulizer solution, calcitriol, calcium acetate, escitalopram, fluticasone nasal spray, ipratropium-albuterol nebulizer, melatonin, midodrine, mirtazapine, pantoprazole, potassium chloride, zinc sulfate, and lorazepam as needed.

On presentation, the patient appeared cachectic and was normocardic but hypotensive to 78/58. Physical examination was remarkable for nontender and nondistended abdomen, and he had 2+ pitting edema in his bilateral lower extremities. Laboratory work-up revealed an acute decline in hemoglobin to 8.7 g/dL from 12.1 g/dL on routine labs drawn two days prior, platelets of 80 x 103/uL, international normalized ratio (INR) of 2.7, serum creatinine of 3.4 mg/dL, and albumin of 1.2 g/dL.

**Figure 1:**
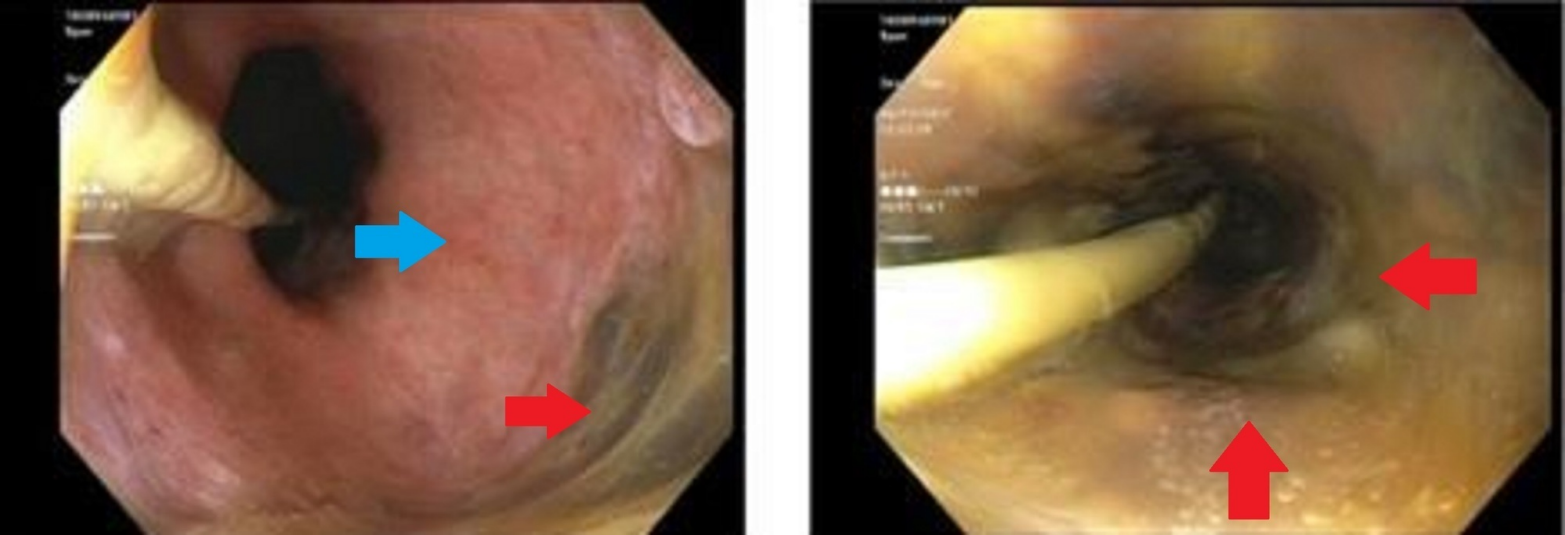
Case 1 on esophagogastroduodenoscopy Distal third of the esophagus with visible necrosis (right, red arrow) and absence of necrosis at the gastroesophageal junction (left, blue arrow).

The patient was administered fluids and was initiated on vasopressors for hemodynamic support. An upper endoscopy revealed a black esophagus extending to the distal third of the esophagus from the GEJ (Figure 1). Erythematous duodenopathy was also noted. The patient received two units of packed red blood cells, maintained on nil-per-os, and started on high dose proton pump inhibitor (PPI) therapy with stabilization of his hemoglobin levels. Despite resolution of his GI bleeding, the patient experienced worsening respiratory status due to the development of bilateral pneumonia with significant parapneumonic pleural effusions requiring mechanical ventilation. He was subsequently transitioned to comfort measures at the request of his family before ultimately passing away.

Case 2

A 54-year-old African American male with a past medical history of alcohol abuse with hepatic steatosis and recurrent pancreatitis presented to the emergency department at an outside hospital hypotensive and tachypneic. Prior to arrival, the patient had been found in a parking lot where he was experiencing multiple episodes of hematemesis and had subsequently become unresponsive. Soon after arrival at the emergency department, the patient suffered cardiac arrest. Return of spontaneous circulation (ROSC) was achieved, and the patient was treated with octreotide and PPI infusion as well as blood transfusion before being transferred to our tertiary care center for further management. Upon transfer, the patient was again found to be hypotensive with systolic pressures in the 50’s and tachypneic. 

**Figure 2:**
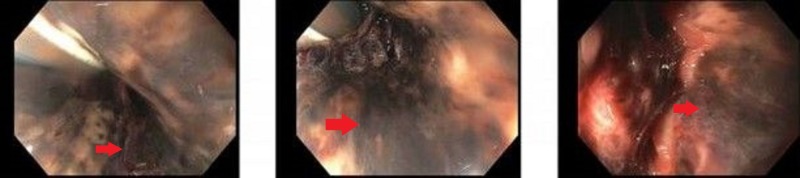
Case 2 on esophagogastroduodenoscopy Middle third of the esophagus displaying diffuse necrosis. (Red arrows pointing towards areas of necrosis).

**Figure 3:**
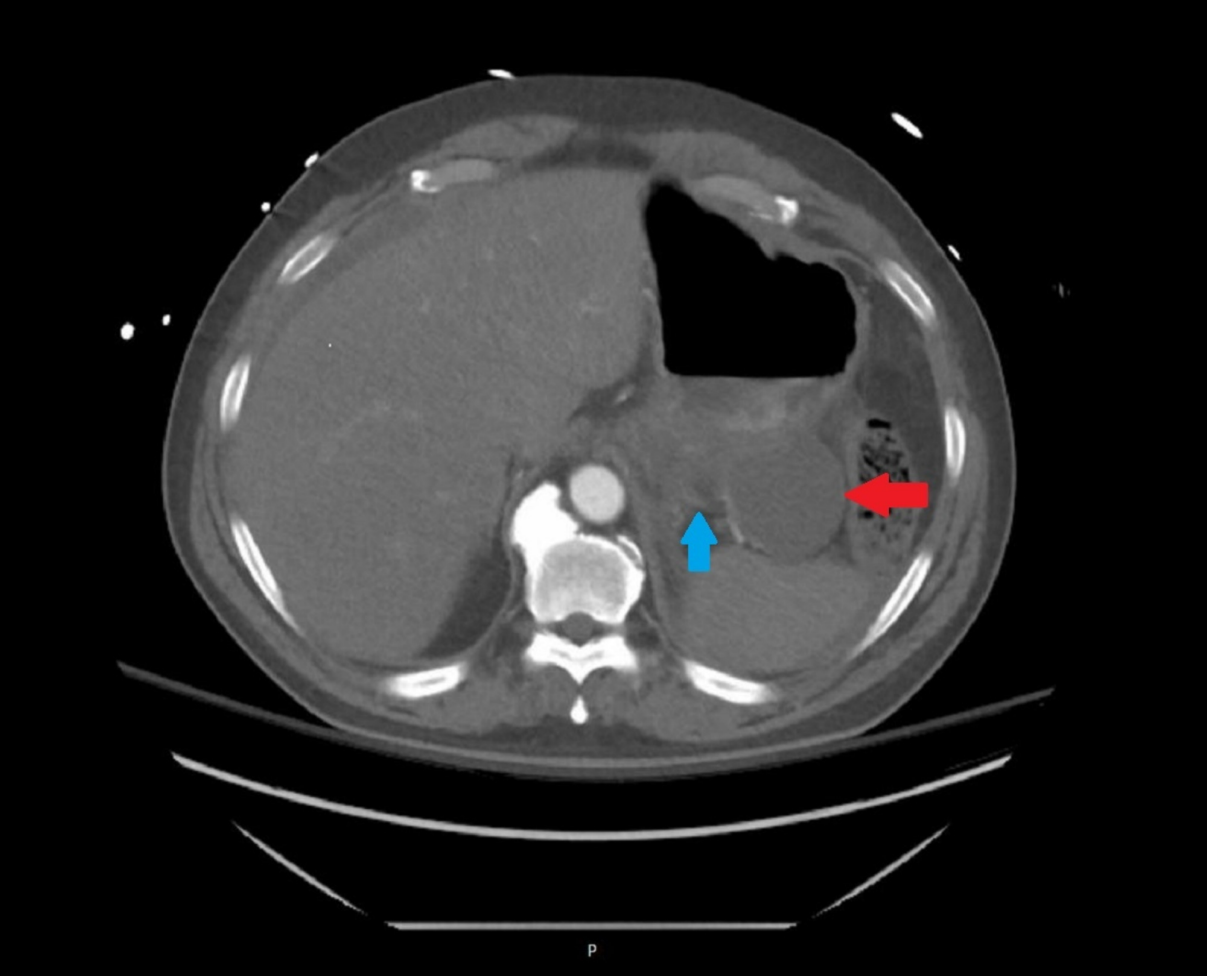
Case 2 CTA Abdomen and Pelvis Patient's CTA abdomen and pelvis showing pseudocyst (red arrow) and general pancreatitis (blue arrow). CTA: Computed tomography angiography

The patient was resuscitated with blood transfusions, intravenous calcium, insulin, dextrose, and prothrombin complex concentrate and vasopressors were started. Laboratory work-up from post-resuscitation revealed hemoglobin of 15.1 g/dL, blood urea nitrogen (BUN) of 61 mg/dL, serum creatinine of 3.5 mg/dL, potassium of 8.2 mmol/L, lactate of 23 mmol/L, and an INR of 1.5. An emergent EGD revealed a black esophagus extending from the GEJ to the middle third of the esophagus with associated diffuse duodenal ulcer disease (Figure 2). No esophagogastric varices were seen. Nil-per-os and high dose PPI therapy were continued and octreotide was stopped. Serum levels of common caustic substances including methanol, ethanol, isopropyl alcohol, acetone, ethylene glycol, and propylene glycol were negative. Computed tomography (CT) of the chest, abdomen, and pelvis revealed severe acute pancreatitis with a 6.4 cm pseudocyst adjacent to the tail (Figure 3). Despite initiation of hemodialysis for persistent hyperkalemia, the patient developed repeated episodes of cardiac arrest and was transitioned to comfort measures upon his family's request. He expired later in the evening.

## Discussion

AEN, with its striking endoscopic appearance of a black esophagus affecting various lengths of the organ and abruptly stopping at the gastroesophageal junction, has received increased recognition over the last decade with frequent endoluminal evaluation of patients presenting with upper gastrointestinal hemorrhage. Its etiology is likely multifactorial – a combination of tissue hypo-perfusion (seen in cardiovascular compromises/shock), massive reflux of gastroduodenal contents (seen in alcohol intoxication and duodenal ulcer disease), and altered mucosal defenses in the esophagus (seen in debilitated states, cancer, and malnutrition). Endoscopic findings are diagnostic and tissue histology is not required but may be supportive. Treatment is aimed at restoring hemodynamic stability and correcting underlying conditions, and includes nil-per-os restriction, blood transfusions, and high dose PPI therapy. Reversibility and restitution of normal appearing mucosa is common. Complications are rare and may include the development of esophageal stenosis or strictures in up to 10% of cases, usually amenable to future endoscopic dilatation. The high mortality rate of nearly 32% is attributed to the severity of underlying clinical conditions. AEN related mortality is low at around 6% [5]. Regrettably, in our cases, even with proper treatment protocol for AEN in place, the severity of the comorbid conditions resulted in the patients’ untimely expiration. 

## Conclusions

Acute esophageal necrosis is a rare, multifactorial manifestation of critical illness. Although it results in a striking endoscopic appearance - black esophagus - treatment should not be aimed at the esophagus itself, but rather at the overarching medical illness causing the patient’s hemodynamic instability. If this is done successfully, patients will have a very high recovery rate.
